# Is there room for children to visit adult intensive care units?

**DOI:** 10.5935/0103-507X.20210020

**Published:** 2021

**Authors:** Silvana Pinto Hartmann, Júlia Schneider Hermel, Cassiano Teixeira

**Affiliations:** 1 Psychology Service, Hospital Moinhos de Vento - Porto Alegre (RS), Brazil.; 2 Internal Medicine Service, Hospital Moinhos de Vento - Porto Alegre (RS), Brazil.

**TO THE EDITOR**

“ My husband has been in the ICU for more than 60 days and I fear that the time for his departure is coming. I was informed of the rule that the visit is allowed from 12 years old, but our daughter is 11. She will be 12 in 2 weeks, but I don’t know if we can wait that long. Could I bring her to say goodbye?”

This request, questioned at the door of an intensive care unit (ICU), causes reflections and generally falls on different opinions and controversies. Brazilian hospitals usually recommend that children under 12 years old do not visit hospitalized patients, given the risks of disease transmission and accidents. Concerns about emotional aspects are also considered. The Brazilian Child and Adolescent Statute considers as a child the person up to 12 years of age incomplete.^(1)^ In ICUs where a Psychology Service is part of the team, requests for a child’s entry are addressed to psychological assessment.^(2)^

A cross-sectional study with 446 North-American adult ICU nurses assessed their perceptions and practices regarding children visits. This research showed that 67.9% of them understood that children were at risk of psychological trauma when visiting an adult in the ICU. Regarding visitation policies for children, 27.4% informed that their ICUs did not have them. The analyses allowed the inference that nurses with a Master’s degree were 1.8 times more likely to believe that children up to 5 years old could visit patients. The chance that nurses would allow children to visit was greater if the patient was the child’s father or if he was dying. The respondents signaled that ICUs could benefit from experts in childhood to facilitate visitation.^(3)^

It should be considered that children play roles within families, whether they are sons/daughters, siblings, nephews, grandchildren, among others, and will continue even if they have critically ill family members. It is usual to hear that “ICU is not a child’s place”, in addition to questions about how would they understand the situation and behave or react to seeing the patient.

By stating that ICU is not a child’s place, the discussion is reduced to the denial of reality. The question could be in the sense of how can one make it possible for the ICU to allow a farewell between a child and a parent? Are catheters, hemodialysis machines, and infusion pumps have more overwhelming than death itself? How can a child be helped to understand the limitations he/she will face in the family regarding his mother’s physical dependence and chronic illness? What can the removal of the child of a puerperal woman with Guillain-Barré syndrome emotionally represent for the patient and the baby?

**Figure 1:**
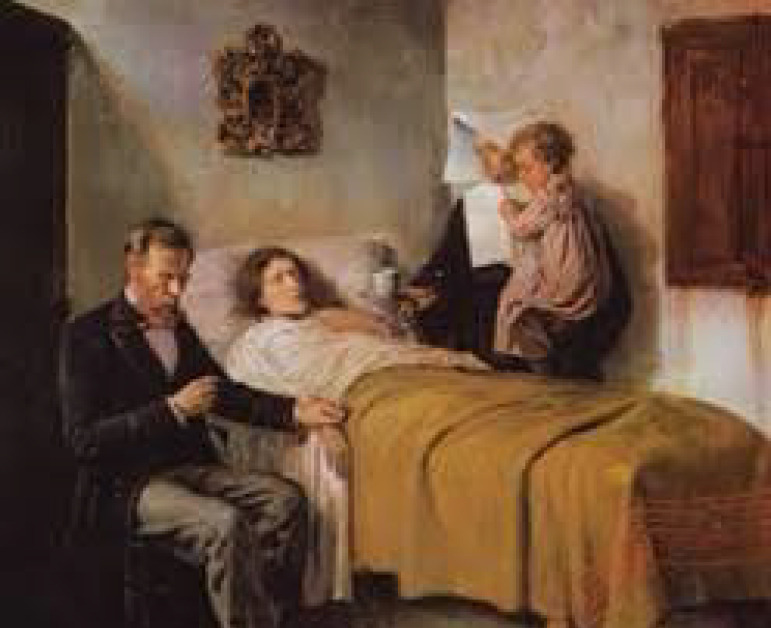
Painting “Science and Charity”, 1897, by Pablo Picasso.

Understanding how children develop the concept of illness may be initially interpreted from a cognitive and cultural point of view. Regarding culture, it is quickly possible to exemplify by observing the painting “Science and Charity”,^(4)^ 1897, by Pablo Picasso, exhibited in the Picasso Museum, Barcelona, Spain (Figure 1). In this painting, one can infer a severely ill woman in the presence of a doctor, a nun, and a child - perhaps the ill woman’s daughter. Ariès^(5)^ justifies that “until the 18^th^ century there is no representation of a dying person’s room without some children”.

Oil on canvas, 197 x 249cm. Barcelona; Picasso Museum.

With the absence or scarcity of hospitals, births, treatment of the sick and death took place at home. Children watched and were part of the reality, exposed as it was. Even today one may hear people who recount the memory of their mothers’ death during childbirth, at home. Certainly, these stories refer to the trauma of losing the mother and the scene they experienced. Today, with deaths in hospitals and ICUs, children are protected from these terrible images, however, the loss is still there. What can be proposed is that there are considerations about the poles in which, culturally, the society is imbued. We’ve emerged from a wide-open reality to the exclusion of the child’s participation in contexts of absences and losses related to family illnesses. What we suggest in these notes is to have proposals for dialogue and ways of elaborating the current context.

Concerning doubts about the child’s understanding of reality, it is required to understand the particularities of each stage of development. For younger than one-year-old babies, contacting the pediatrician regarding the baby’s health status and exposure to a critical environment. Children between zero and 2 years old know the outside world through their senses and motor skills. Children under 2 years perceive the absence and miss their parents or caregivers. Children between 3 and 5 years understand the world through individual and egocentric perspectives. Death is seen as a temporary and reversible phenomenon. Between 6 and 9 years, children have concrete thinking and have notions of irreversibility. They can be encouraged to ask questions about the visits. From 10 years old until adolescence, they fully understand the situation they find themselves in as well as death’s irreversibility. Even for younger and more independent children, it is necessary to support and provide precise information about the patient.^(6,7)^

Knowing this, the healthcare team, when approached regarding a visitation request, should identify whose desire is the visit.^(2)^ Depending on the age, it can be the child’s will, and, therefore, it is imperative to know what was the child told about the family member’s hospitalization. In routine practice, difficulties are identified in these aspects. There are reports of silences and use of metaphors, aiming to mitigate the suffering. Sometimes it is identified that the visitation request would be a way to make someone from the healthcare team communicate to the child something the family feels difficult to do. However, the healthcare professionals should not take the responsibility for a dialogue that belongs to the families, however, they can assist them and provide instruments. It is also necessary, whenever possible, to have the patient’s agreement.

The ICU’s team must be consulted in a multidisciplinary discussion held on each cases’ viability. The child must be accompanied by a guardian with whom he or she has an affective bond. This family member should also reflect on how would feel about the visit, as it will be his/her responsibility to communicate and support the child.

If there is a consensus of all parties, ways must be used to assure the child is protected from the ICU surroundings, keeping the patient covered, neighbor patients’ curtains kept closed, or box doors kept close if the ICU has individualized beds. The visitation is recommended to be short, for a few minutes.^(6)^ It should be considered that intercurrences are unpredictable, therefore the time of the visitation should be upon multidisciplinary agreement. When possible, one could wait for the patient’s discharge from intensive care, and the visit held at the ward.

**Figure 2:**
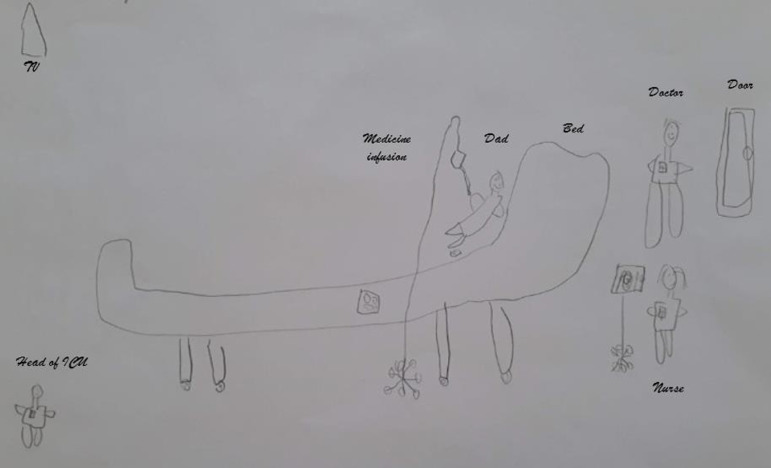
Child’s drawing.

As an example, upon a visitation request, the psychologist interviewed the mother of a 7-year-old child who manifested willingness that her son could visit his postoperative father at the ICU. Questioning the child’s understanding, the psychologist asked the boy to draw how did he imagine his father would be (Figure 2). The boy explained his drawing: his father was laying in his bed “taking medicine in the vein”. By his side, a table for meals, and at the same side, the nurse and the doctor. In front of him, the TV set and the ICU chief. After the visit, a new approach was made to assess the adequacy of the reality imagined and the boy’s experience. He said that found his father better than expected, as he was seated on an armchair and not laying on the bed as he imagined.

Authorized.

Concerns about visiting children are legitimate and highlight the need of establishing a routine in ICUs. In the context of pandemics, where restrictions are worldwide recommended, the risks for people circulating in hospitals are undisputable. To mitigate the distance between patients and their families, currently, electronic devices such as computers and cell phones can provide virtual video call visitations. In this case, the risk of infections is controlled. But regardless of the type of visitation, the topics herein discussed should continue to be considered as related to children, in addition to chronological milestones.

This discussion is proposed to evaluate the possibility of visits with criteria and care. The visitation, when its context is known and held safely, can be subjectively relevant for the families, and necessary for the elaboration of absences and missings caused by illness.
